# Effect of Activated Charcoal Fibers on the Survival of the House Dust Mite, *Dermatophagoides pteronyssinus*: A Pilot Study

**DOI:** 10.5402/2012/868170

**Published:** 2012-10-24

**Authors:** Hae-Seon Nam, Sun-Hwa Lee, Young-Jin Choi, Joon-Soo Park, Moon-Kyun Cho, Sang-Han Lee, Julian Crane, Robert Siebers

**Affiliations:** ^1^Division of Infectious Diseases and Allergy, Soonchunghyang Medical Research Institute, Soonchunhyang University, Cheonan 330-090, Republic of Korea; ^2^Department of Clinical Parasitology, Soonchunhyang University, Cheonan 330-090, Republic of Korea; ^3^Department of Dermatology, Soonchunhyang University, Cheonan 330-090, Republic of Korea; ^4^Department of Biochemistry, School of Medicine, Soonchunhyang University, Cheonan 330-090, Republic of Korea; ^5^Wellington Asthma Research Group, School of Medicine and Health Sciences, University of Otago, Wellington 6242, New Zealand

## Abstract

House dust mites produce potent allergens that exacerbate asthma in sensitized patients, whom are recommended to practice allergen avoidance within their home environment. We tested the effect of activated charcoal impregnated fibers on house dust mite survival. One hundred live adult house dust mites (*Dermatophagoides pteronyssinus*) were added to eight culture dishes preequilibrated at room temperature (*n* = 4) and 70% humidity (*n* = 4) containing house dust mite food and active charcoal fibers. At 10 minute intervals, live and dead house dust mites were counted. All house dust mites instantly attached to the activated charcoal fibers and started to shrink almost immediately. There were no live house dust mites present as early as 40 minutes in some dishes while after 190 minutes all house dust mites were dead. In conclusion, activated charcoal fibers, if incorporated into bedding items, have the potential to control house dust mites in the indoor environment.

## 1. Introduction

House dust mites (HDMs) produce potent allergens that can exacerbate asthma in sensitized patients [1]. HDM sensitized patients are generally recommended to practice allergen avoidance within their home environment, especially in the bedroom where they spend about one-third of their lives in close proximity to allergen-laden bedding items [2]. Commonly recommended HDM avoidance methods include covering all bedding with HDM impermeable materials. Although various studies have shown clinical efficacy of occlusive covers [3, 4], a recent large trial concluded they were ineffective [5]. Additionally, a recent systematic review has concluded that chemical and physical methods for reducing HDM allergens are not recommended [6]. However, this study has been criticized regarding its inclusion and exclusion criteria and evaluation of the studies [7]. 

Occlusive bedding covers, recommended by allergy and asthma societies worldwide, are expensive and alternative methods have been sought to reduce exposure to HDM allergens in bedding. We have previously shown that activated charcoal powder added to HDM food suppresses breeding of the HDM, *Dermatophagoides pteronyssinus* in the laboratory setting thus offering a potential new method for HDM control [8]. However, activated charcoal powder is messy and difficult to incorporate into bedding items. We therefore tested the effects of fibers impregnated with activated charcoal on HDM survival.

## 2. Methods

To each of eight culture dishes, diameter 50 mm, height 15 mm (Corning, USA), 0.02 g of HDM food consisting of wheat germ (40%), granulated yeast (40%), commercial fish food (15%), and dried daphnia (5%) plus 0.035 g of short cut activated charcoal fibers (Carboflex, Korea Activated Carbon Fiber Ltd., Korea), were added. Four of these dishes (Group A) were equilibrated at room temperature (48–53% humidity and 21–26°C) and the other four in a moisture chamber at 70% humidity and 25°C (Group B). After seven days of equilibration 100 live and active adult *Dermatophagoides pteronyssinus *HDM were added to each dish, covered and then observed under a stereo microscope (Leica, Heerbrugg, Switzerland) at 10 minute intervals for live and dead HDM, which were counted and recorded. Additionally, four dishes with house dust mite food and live house dust mites (as above) but without activated charcoal fibers acted as controls (two at room temperature and two at 70% humidity).

## 3. Results

**Figure 1:**
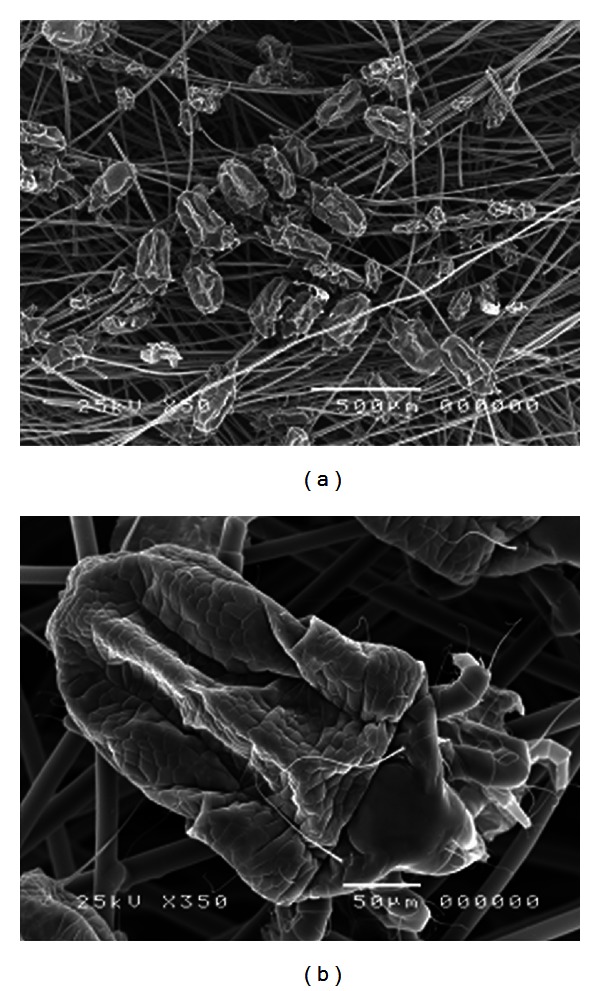
Shriveled house dust mites (*D. pteronyssinus*) in contact with activated charcoal fibers.

All the HDM instantly attached to the activated charcoal fibers and started to shrink almost immediately (Figure 1). There were no live HDM present in some dishes as early as 40 minutes after addition.  Table 1 shows that the average percentage of dead HDM was 95% or greater after 60 minutes while after 190 minutes we observed that no dish had any live HDM present (data not shown). In the control group all added HDM were still alive at 140 minutes and had only slightly reduced at 24 hrs, 48 hrs, and 72 hrs (data not shown).

## 4. Discussion

This study has shown that upon contact with activated charcoal fibers, HDM shrink almost immediately and within a relative short time period no live HDM could be observed. This effect is likely due to the activated charcoal fibers as without their addition to the dishes the HDM stayed alive for a prolonged period. The most likely mechanism for the killing effect of activated charcoal fibers is through absorption of moisture from HDM. Activated charcoal fibers are widely used in industry due to their great adsorption properties and desirable physical properties, such as high mechanical strength, thermal resistance, light weight, and safety profile [9]. Industrial and commercial uses include water treatment and gas masks and equipment for removal of toxic gases. In Korea some bedding manufacturers incorporate activated charcoal in pillows with unsubstantiated health benefits, including control of asthma symptoms.

A limitation of the study is that only one concentration of activated charcoal fibers was used. Studies on diminishing concentrations of activated charcoal fibers until no mite deaths are observed would be required to give an idea of its potency. Furthermore, the killing effect may not have been due to the activated carbon and possibly due to some other contaminant on the fibers. Studies are required to see whether uncoated fibers have a similar effect. However, the effect of the of the activated charcoal fibers was direct and striking (Figure 1) and strongly suggests that it was due to moisture extraction from the house dust mites.

In conclusion, activated charcoal fibers incorporated into bedding items have the potential to control HDM in the indoor environment. Further research is required on how to incorporate these fibers in bulkier bedding items, such as mattresses and duvets. Also, as bedding occupants produce great amounts of moisture during sleep, the long-term effectiveness of activated charcoal fibers incorporated into bedding items to continue to absorb moisture needs to be determined and how to regenerate the fibers after complete saturation. Finally, well controlled clinical trials are required to demonstrate its effectiveness in reducing asthma symptoms, quality of life, and asthma medication use.

## Figures and Tables

**Table 1:** Percentages of dead house dust mites in the presence of activated charcoal fibres.

	10 min	20 min	30 min	40 min	50 min	60 min	70 min
A*	48.2% (41–57)	71.5% (50–82)	91.5% (82–96)	94.5% (86–100)	95.5% (87–100)	97.0% (91–100)	97.5% (92–100)
B**	25.8% (18–30)	53.5% (45–61)	80.8% (71–92)				

*A: equilibrated at room temperature (48–53% humidity and 21–26°C).

**B: equilibrated in moisture chamber at 70% humidity and 25°C.

Results are means with range of values in parentheses.
